# Septic Arthritis Caused by *pantoea agglomerans* in an Patient Without Known Immunosuppressive Conditions: A Case Report

**DOI:** 10.1002/ccr3.72721

**Published:** 2026-05-12

**Authors:** Paul Ngongo Tshonda, Delphin Murhula Katabana, Roland Lwandiko, Yannick Chibinda Birato, Djibril Mutawa, Pascal Murhula, Rodrigues Mupenda Mwenibamba, Tony Akilimali Shindano

**Affiliations:** ^1^ Department of Internal Medicine Bukavu University Clinics Bukavu South Kivu Democratic Republic of Congo; ^2^ University of Kindu Kindu Democratic Republic of Congo; ^3^ Department of Surgery Bukavu University Clinics Bukavu South Kivu Democratic Republic of Congo

**Keywords:** case report, osteoarticular infection, *Pantoea agglomerans*, septic arthritis

## Abstract

Septic arthritis caused by *Pantoea agglomerans* is uncommon in immunocompetent patients. This case emphasizes the importance of considering atypical pathogens in septic arthritis, underscores the role of microbiological confirmation, and highlights the necessity of prompt, targeted antimicrobial therapy to achieve favorable outcomes.

## 1 Introduction

Septic arthritis is a rare but serious condition, with prognosis largely dependent on early diagnosis and prompt treatment [1, 2]. It is most commonly caused by *Staphylococcus aureus* , streptococci, or other pyogenic bacteria [1]. In certain clinical contexts, however, opportunistic or environmental pathogens such as *Pantoea agglomerans* ( *P. agglomerans* ) may be implicated [3, 4]. This enterobacterium, typically found in soil and associated with plants, is rarely responsible for human infections and only exceptionally for septic arthritis [4, 5, 6]. We present a case in an immunocompetent patient successfully managed at Bukavu University Clinics in eastern Democratic Republic of the Congo.

## 2 Case History/Examination

A 71‐year‐old male professional driver presented in February 2025 with progressive right knee pain that had developed over several months. Initially mechanical in nature, the symptoms gradually evolved into an inflammatory pattern, prompting medical consultation. His medical history included hypertension, managed with amlodipine and telmisartan, and benign prostatic hypertrophy, treated with Tamsulosin. He had no known debilitating conditions, history of trauma, or prior invasive joint procedures. On further questioning, he reported recent gardening activity. He was a non‐smoker, did not consume alcohol, and denied use of immunosuppressive drugs or prior complaints suggestive of chronic inflammatory arthritis such as gout or rheumatoid arthritis.

**FIGURE 1:**
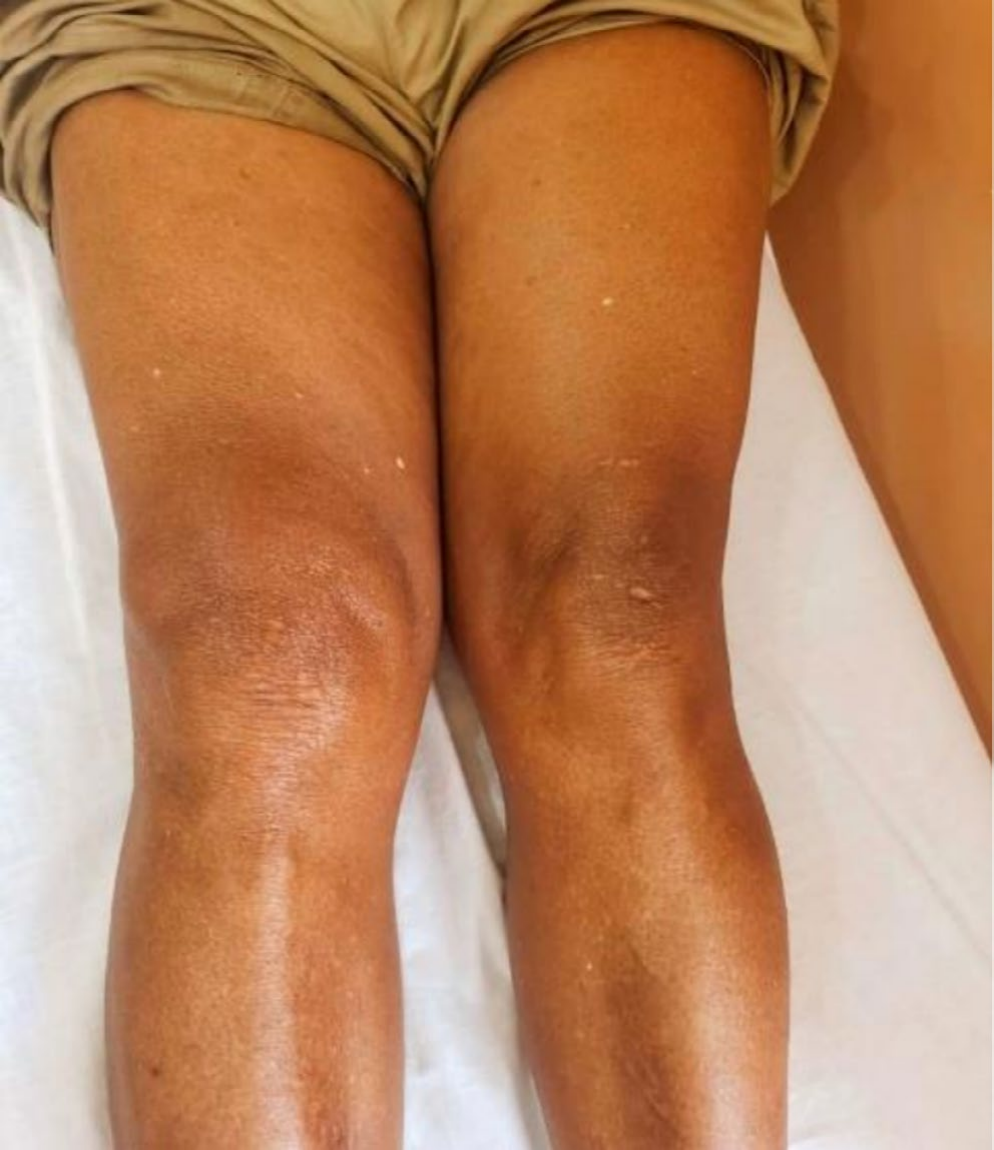
Picture of the right knee before joint aspiration, showing increased swelling compared to the left knee.

**FIGURE 2:**
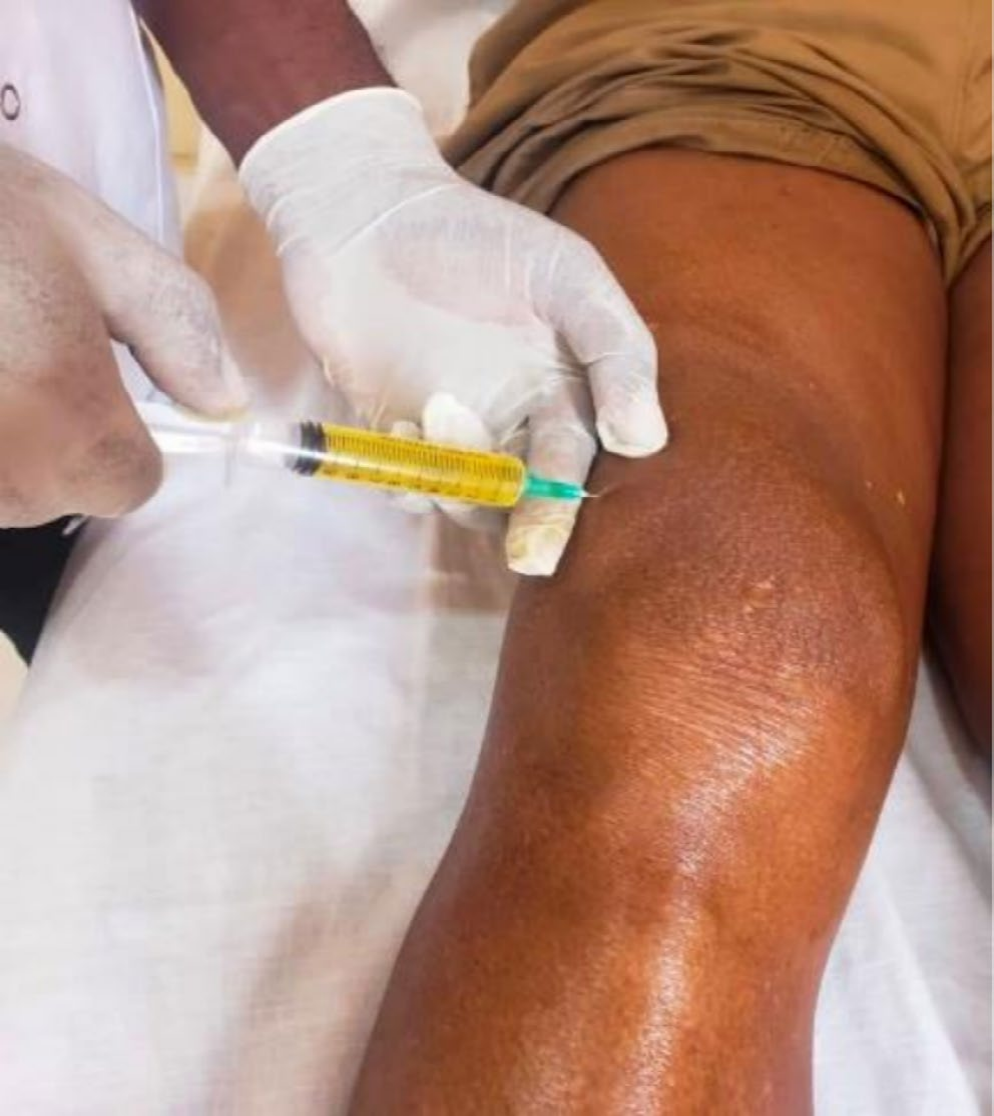
Image of the joint aspiration procedure during which a yellow and turbid synovial fluid was collected.

On admission, the patient was in good general condition, with a temperature of 36°C, blood pressure of 133/104 mmHg, and heart rate of 104 bpm. Examination of the right knee revealed swelling (Figure 1), warmth, and a positive patellar shock, with moderately reduced range of motion. Laboratory investigations showed a C‐reactive protein (CRP) level of 20 mg/L, erythrocyte sedimentation rate (ESR) of 40 mm/h, uric acid of 4 mg/dL, and a normal white blood cell count (4130/mm^3^, with 66.3% neutrophils). Random blood glucose was 110 mg/dL, and fasting glucose was 98 mg/dL. Joint aspiration yielded turbid yellow fluid (Figure 2), which was sent for culture.

**FIGURE 3:**
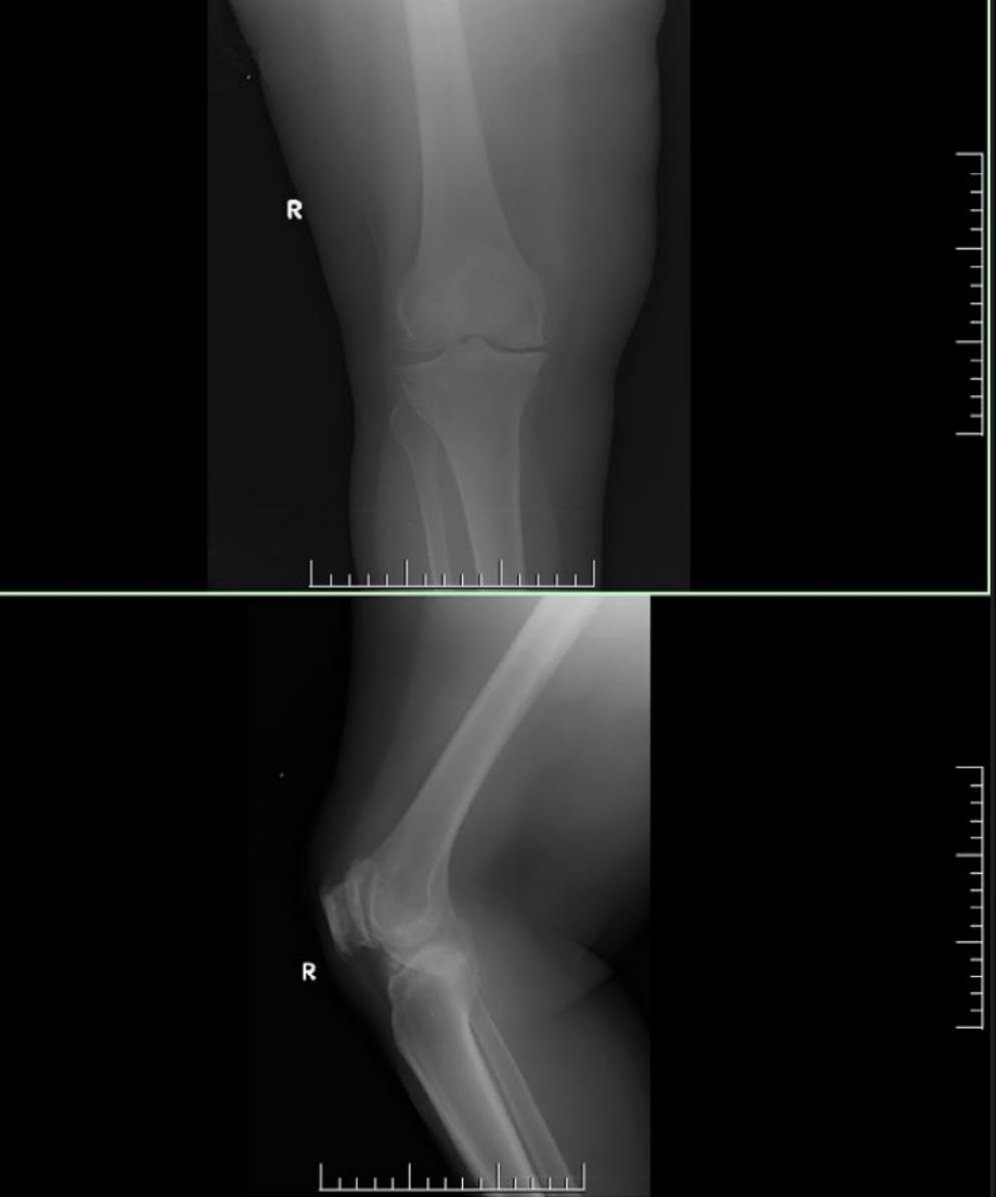
Radiographic image of the right knee, demonstrating reduced joint space.

X‐ray imaging showed joint space narrowing (Figure 3). MRI and arthrography were unavailable locally. Pending culture results, empirical therapy with amoxicillin–clavulanic acid, ibuprofen, and a proton pump inhibitor was initiated.

Joint culture identified *P. agglomerans* , with antibiogram results showing sensitivity to piperacillin–tazobactam, amikacin, gentamicin, tobramycin, aztreonam, ticarcillin, and mycocycline. The isolate was resistant to several β‐lactams (cefotaxime, cefepime, cefazolin, ceftazidime), ciprofloxacin, and chloramphenicol. This prompted an HIV rapid test (Determine), which was negative. Targeted therapy was initiated with piperacillin–tazobactam (2 × 4.5 g/day) and amikacin (2 × 500 mg/day), combined with etoricoxib. Joint drainage and immobilization with a plaster cast were performed concurrently. After 5 days, serum creatinine increased from 1.3 to 2.1 mg/dL, necessitating discontinuation of amikacin. The patient remained hemodynamically stable, with gradual improvement in knee pain and swelling. On day 10 of intravenous therapy, CRP had decreased to 3 mg/L and ESR to 20 mm/h. Intravenous piperacillin–tazobactam was continued until Day 21.

## 3 Differential Diagnosis

Differential diagnoses included crystal‐induced arthritis (gout or pseudogout), degenerative osteoarthritis flare, and inflammatory arthritis. Normal uric acid levels, absence of crystals, inflammatory synovial fluid, and a positive bacterial culture excluded these possibilities and confirmed septic arthritis.

## 4 Conclusion and Results

**FIGURE 4:**
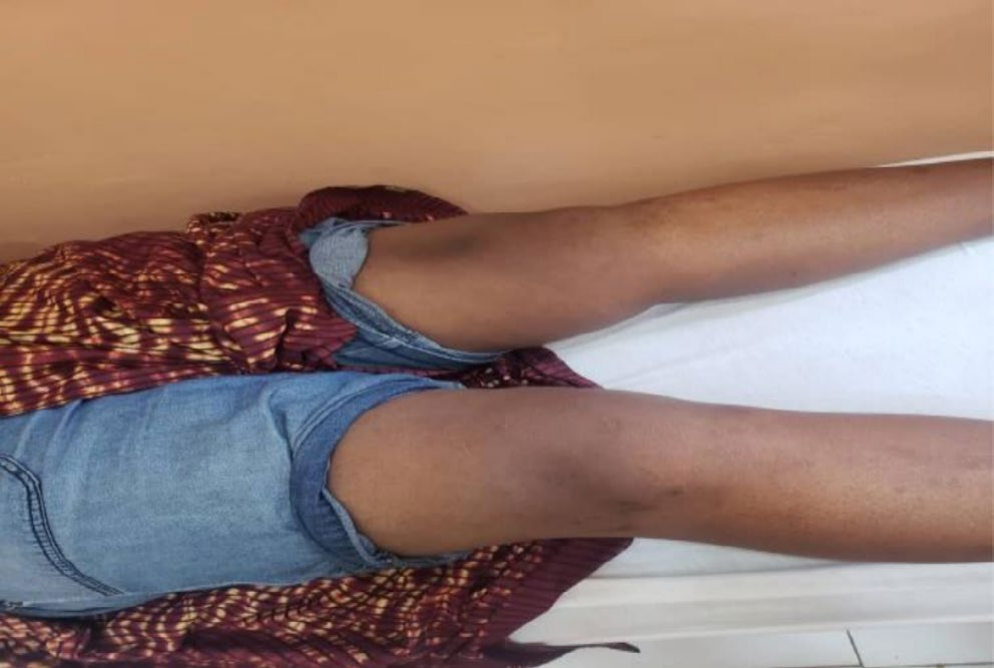
Picture of the right knee 1 month after completion of treatment, showing resolution of swelling and inflammatory signs.

After 21 days of targeted intravenous antibiotic therapy, the patient demonstrated significant clinical and biological improvement. Knee pain and swelling progressively resolved, and inflammatory markers normalized by the end of treatment. At 1 month (Figure 4) and three‐month follow‐up visits, the patient remained clinically stable, with no evidence of recurrent infection. Physical examination revealed normal joint mobility, absence of effusion, and no local inflammatory signs. The only residual symptom was mild knee pain during stair climbing, without functional limitation.

## 5 Discussion

Septic arthritis is a medical emergency, requiring rapid treatment to prevent serious complications such as permanent joint damage. It is most frequently caused by saprophytic skin bacteria, including *Staphylococcus aureus* and *Streptococcus* species [1, 2].

*P. agglomerans* is a Gram‐negative member of the Enterobacteriaceae family, originally described in environmental contexts [7, 8, 9, 10, 11]. Human infections are rare, with clinical presentations ranging from bacteremia [12] and abscesses to respiratory or urinary tract infections, and occasionally septic arthritis, typically in immunocompromised patients or following plant‐related trauma [1, 5, 13, 14].

Reports of septic arthritis due to *P. agglomerans* are scarce, generally involving male patients with a predilection for the knee joint [5, 6]. Most cases occur after plant puncture injuries or invasive procedures [5, 9, 15]. In the present case, unnoticed microtrauma during gardening is the most plausible source of infection. Although no classical immunosuppressive condition was identified, the patient's advanced age (71 years) may have predisposed him to opportunistic infection through immunosenescence and age‐related immune dysfunction [16]. Immunosenescence is characterized by thymic involution, reduced production and function of T and B lymphocytes, impaired innate immunity, and chronic low‐grade inflammation, all of which increase susceptibility to infection even in the absence of overt immunosuppressive disease [10, 16]. Furthermore, aging is associated with a blunted inflammatory response, and fever may be absent or less pronounced in elderly patients with infection [10], which may explain the lack of fever in this case.

Another possible explanation involves the local joint environment. The most plausible hypothesis is septic arthritis secondary to discrete local inoculation on pre‐existing arthritic tissue. The initial presentation was mechanical, consistent with underlying osteoarthritic pain, before progressing to an inflammatory syndrome. Osteoarthritis, primarily a degenerative cartilage disease, may evolve into congestive synovitis and compromise local joint defense mechanisms, thereby facilitating bacterial invasion [17].

Diagnosis is typically established through joint fluid culture, with bacteriological identification and antibiogram [1, 2]. Treatment relies on targeted antibiotic therapy, often combined with joint drainage (puncture, lavage, or surgery). In published cases of septic arthritis due to *P. agglomerans* , broad‐spectrum β‐lactams have frequently been used empirically, as reported by Koutserimpas et al. [5]. Our patient was initially treated with amoxicillin–clavulanic acid, followed by piperacillin–tazobactam and amikacin, guided by antibiogram results and suspicion of septic origin. Aminoglycosides, however, are associated with nephrotoxicity, particularly in elderly patients [18]. In this case, serum creatinine increased after 5 days, necessitating discontinuation of amikacin. Renal function subsequently stabilized, underscoring the importance of close monitoring and timely adjustment of therapy when aminoglycosides are used. Exclusive medical management achieved satisfactory outcomes: after 21 days of treatment, joint fluid collection had resolved, inflammatory signs markedly decreased, and follow‐up evaluations at 1 and 3 months revealed no recurrence.

This case raises pathophysiological questions regarding the septic origin of the lesion in a patient without obvious predisposing factors. Hematogenous spread appears unlikely in the absence of active infection or another entry point. The patient denied trauma or invasive procedures, but recent gardening suggests the possibility of unnoticed microtrauma, facilitating inoculation of the pathogen.

Some diagnostic limitations should be acknowledged. Advanced imaging modalities such as magnetic resonance imaging or computed tomography were not available in our setting. In such situations, musculoskeletal ultrasound can be particularly useful for detecting joint effusion and guiding joint aspiration when septic arthritis is suspected [19, 20]. In addition, Gram stain examination, synovial fluid cell count, and biochemical analysis were not performed due to laboratory constraints. Despite these limitations, the turbid yellow appearance of the synovial fluid and the identification of *P. agglomerans* on culture allowed confirmation of the diagnosis.

To date, approximately 15 cases of septic arthritis caused by *P. agglomerans* have been reported in the literature [5]. The majority involved male patients (> 80%), with predominant knee involvement (> 75%), consistent with our case [5]. The mean age of reported patients was 30.5 ± 24 years, making our patient the oldest described to date. What distinguishes this case is that nearly all previous reports followed percutaneous trauma, medical procedures, or debilitating conditions. Although similar cases in immunocompetent patients have been documented [21, 22], they remain rare. This report contributes further clinical evidence and underscores the importance of considering atypical pathogens in septic arthritis, even in patients without obvious risk factors.

Our patient responded favorably to medical therapy alone, whereas surgical debridement is often performed to exclude foreign bodies contributing to infection. A 21‐day course of antibiotics eradicated all clinical and biological signs of infection. In published cases, longer treatment durations were sometimes required, with a mean of 52 days (range 21–106), particularly when intra‐articular debris was present.

Cases of septic arthritis caused by atypical or unusual microorganisms have been increasingly reported in the literature. In addition to *P. agglomerans* , other atypical pathogens such as *Pseudomonas aeruginosa* or *S. xylosus* have been described in recent reports of osteoarticular infections, including rare localizations such as the shoulder or sacroiliac joint [23, 24]. This highlights the importance of broadening the etiological spectrum and paraclinical investigations when evaluating septic arthritis, particularly when the clinical presentation is atypical.

## 6 Conclusion

This case demonstrates septic arthritis due to *P. agglomerans* in a patient without classical immunosuppressive risk factors, and in the absence of trauma or identified plant exposure. It underscores the importance of systematically culturing joint fluid when septic arthritis is suspected, and the value of rapid, targeted therapy guided by antibiogram results. Recognition of atypical pathogens enables optimized management and reduces the risk of long‐term functional sequelae.

## Author Contributions

**Paul Ngongo Tshonda:** conceptualization, investigation, data curation, clinical management of the patient, writing – original draft, writing – review and editing. **Delphin Murhula Katabana:** supervision, validation, clinical management of the patient, writing – review and editing. **Roland Lwandiko:** investigation, writing – review and editing. **Yannick Chibinda Birato:** investigation, literature review, writing – review and editing. **Djibril Mutawa:** investigation, writing – review and editing. **Pascal Murhula:** investigation, writing – review and editing. **Rodrigues Mupenda Mwenibamba:** supervision, validation, writing – review and editing. **Tony Akilimali Shindano:** supervision, project administration, validation, and final approval of the manuscript.

## Funding

The authors have nothing to report.

## Consent

Written informed consent was obtained from the patient for publication of this case report.

## Conflicts of Interest

The authors declare no conflicts of interest.

## Data Availability

Data sharing not applicable to this article as no datasets were generated or analysed during the current study.
